# Protein kinase G oxidation contribute to hypotension and organ injury during sepsis

**DOI:** 10.1186/2050-6511-14-S1-P58

**Published:** 2013-08-29

**Authors:** Olena Rudyk, Alkystis Phinikaridou, Oleksandra Prysyazhna, Joseph R Burgoyne, Rene Botnar, Philip Eaton

**Affiliations:** 1 Cardiovascular Division, King’s College London, London, SE1 7EH, UK; 2 Division of Imaging Science and Biomedical Engineering, King’s College London, London, SE1 7EH, UK

## Background

Sepsis is a medical condition caused by severe infection with systemic inflammation, substantial morbidity and poor survival prognosis. Major clinical features of sepsis include abnormally low blood pressure, decreased peripheral vascular resistance, micro-vascular leak, and decreased cardiac output. Together this leads to inadequate perfusion of vital organs which can result in their necrosis, and often patient death. Multiple oxidant-generating systems contribute to increased level of oxidants, such as hydrogen peroxide, peroxynitrite and nitrosothiols during sepsis. Previously we demonstrated that oxidants can activate PKGIα to lower blood pressure. In a current study we hypothesised that oxidative activation of PKG underlies sepsis-induced hypotension and consequential organ injury.

## Materials and methods

We employed a “redox-dead” Cys42Ser PKGIα knock-in (KI) mouse which cannot be oxidant-activated. Experimental endotoxemia by intraperitoneal injection of the bacterial endotoxin lipopolysaccharide (LPS, 9mg/kg) and a cecal ligation and perforation (CLP) model of sepsis were studied.

## Results

Peak hypotension (ΔMAP 19±4 mmHg in WT vs 6±3 mmHg in KI) caused by endotoxemia was 3-fold greater in WT than in KI mice. PKGIα oxidation (assessed by disulfide dimerization using immunoblotting) was increased in WT mesenteric vessels and cardiac tissue compared to vehicle-treated controls after LPS treatment. KI mice also were protected from CLP-induced sepsis during initial hypotension peak (ΔMAP 22±5 mmHg in WT vs 10±3 mmHg in KIs) and over the entire observation period. CLP, like LPS, also increased PKGIα oxidation in WT mesenteries and cardiac tissue compared to controls. The maximal U46619-induced vasoconstriction in WT non-septic vessels was 10.0±0.9 mN, whereas sepsis reduced this to 8.3±0.2 mN; however, sepsis did not lower the constriction in KI, which is consistent with their protection from hypotension *in vivo*. CLP significantly increased vascular leak 8- and 4-fold in WT mesenteries and aorta respectively compared to vessels from KI littermates. Cardiac function was better in septic KI mice, with a preserved stroke volume and cardiac output relative to WT. Notably, KI mice subjected to CLP had lower plasma blood urea nitrogen and lactate dehydrogenase levels, suggesting less renal and global tissue damage compared to WT. Moreover septic KIs were characterised by attenuated hypothermia and preserved locomotor activity indicating better systemic well-being compared to WT.

## Conclusion

Sepsis, long associated with oxidative stress, induces disulfide-activated PKGIα to lower blood pressure. This hypotension results in under-perfusion of end organs and systemic dysfunction. These findings provide new insight for rationale therapy design, for example drugs that limit PKGIα disulphide formation may be protective.

